# A novel class-attention transformer-driven feature fusion technique-based speech disorder classification

**DOI:** 10.3389/fmed.2026.1812143

**Published:** 2026-06-23

**Authors:** Abdul Rahaman Wahab Sait, Haitham Ahmed Jamil Mohammed, Taqwa Ali Mohammad Bani Awad, Ali Mohammad Alorsan Bani Awad

**Affiliations:** 1 Department of Documents and Archive, Center of Documents and Administrative Communication, King Faisal University, Al-Ahsa, Saudi Arabia; 2 Faculty of Computer Science and Information Technology, Elimam Elmahdi University, Kosti, Sudan; 3 Princess Basma Teaching Hospital, Irbid, Jordan; 4 Royal Medical Services, Amman, Jordan; 5 Quality Assurance and Accreditation Unit, Deanship of Development and Quality Assurance, King Faisal University, Al-Ahsa, Saudi Arabia

**Keywords:** deep learning, feature fusion, model interpretability, Parkinson’s disease, speech disorder classification, vision transformer

## Abstract

**Introduction:**

Speech disorders (SD) present significant diagnostic challenges due to the complex and indistinct acoustic characteristics embedded within speech signals.

**Problem statement:**

The standalone convolutional neural networks (CNNs) and vision transformers (ViT) struggle to capture SD temporal and spectral dynamics.

**Objectives:**

To address these limitations, this study proposes an end-to-end binary pathological SD detection framework that processes raw audio waveforms to differentiate disordered speech from healthy speech while improving feature representation, interpretability, and generalization.

**Methods:**

A hybrid feature extraction integrating one-dimensional CNNs and ViT’s self-attention mechanism is developed to extract crucial SD features. An adaptive fusion and a Class-attention transformer (CaiT)-based feature refinement is introduced to transform the extracted features into a classification-optimized global representation. Through the integration of gradient-weighted class activation mapping (Grad-CAM) and attention-based visualization with the model architecture, the temporal-localization of disorder-relevant acoustic patterns is enabled. Two benchmark datasets are utilized for model’s performance evaluation, benchmarking against state-of-the-art CNNs and ViT architectures. The model is trained and internally validated on the SVD dataset using subject-level splitting, while the PD and VOICED datasets are used exclusively for external validation to assess cross-dataset generalization across neurological and heterogeneous pathological voice conditions.

**Results:**

The proposed model achieves 97.50% accuracy on both SVD and PD datasets and 95.80% accuracy on VOICED, while maintaining a lightweight design with 5.2 million parameters. Statistical analysis further confirms the results’ reliability and significance.

**Conclusion:**

The integration of adaptive fusion and transformer-based refinement significantly enhances SD detection performance while ensuring interpretability. The suggested framework offers a sound and proof-of-concept for diagnosing SD, allowing clinicians and speech-language pathologists to make reliable predictions and pay attention to diagnostically significant time intervals.

## 1 Introduction

Speech disorders (SD) comprise a broad spectrum of conditions, including Parkinson’s disease (PD), amyotrophic lateral sclerosis, vocal fold paralysis, dysarthria, and apraxia, affecting individuals’ communication and quality of life (1–3). These disorders are characterized by unique vocal abnormalities that frequently overlap, posing substantial diagnostic challenges (4). In order to initiate appropriate therapies, improve therapeutic results, and alleviate patient suffering, there is a demand for early detection and precise categorization of these SD. With its low cost and scalability, biomarkers produced from acoustic analysis offer a promising non-invasive diagnostic approach (5). However, ambient noise, individual variability, subtle inter-disorder variations, and substantial symptom overlap cause challenges in differentiating the disorders using acoustic biomarkers (5–7).

Traditionally, the SD identification primarily relies on acoustic features, including jitter, shimmer, harmonics-to-noise ratio, pitch perturbations, and Mel-frequency cepstral coefficients (8). Extraction of these features is a standard procedure among machine learning approaches, such as Gradient Boosting Machines (GBMs), Support Vector Machines (SVM), Random Forests, and Extremely Randomized Trees (ExtraTrees) for SD classification (9–11). However, these approaches have a number of intrinsic limitations due to their architecture. Handcrafted features are unable to highlight complex, high-dimensional spectro-temporal acoustic patterns associated with speech impairments, reducing the SD detection model’s generalizability across diverse datasets and clinical settings (12–14). Furthermore, traditional classifiers exhibit limited adaptability, leading to sub-optimal performance in a real-time environment (14).

Machine learning and deep learning are gaining prominence in shaping contemporary healthcare, enabling the discovery of multidimensional associations in high-dimensional clinical data (15). These models are applied in the field of neuroscience to interpret speech as an organized signal of motor control, thought processes, and neural coordination (16–18). The differences in speech production, including inconsistencies in timing, phonatory stability, and articulation dynamics, are related to impairments in neural pathways. Machine learning and deep learning models train on these complex patterns and project them to a particular brain state (19–21). This supports clinicians in making more accurate, data-driven decisions and helps increase the efficiency of neurological care by facilitating automated screening, objective severity assessment, and longitudinal tracking.

Recent studies reveal the potential of deep learning approaches in the field of acoustic modeling (22–24). In comparison with traditional machine learning methods, Convolutional Neural Networks (CNN) and Vision Transformers (ViT) have shown remarkable improvement in SD classification (24). CNNs effectively extracts local auditory characteristics directly from spectrogram representations, representing rich and complicated sound patterns (25). They are ideal for the SD detections due to their ability to successfully capture subtle, disorder-specific characteristics, including transitory amplitude disturbances or micro-tremors (26). However, CNNs’ fixed-size receptive fields restrict their capacity to capture global speech signal correlations (26). They are unsuitable for reflecting speech impairments with long-term temporal or spectral dependence (26).

ViTs have gained prominence due to their ability to use self-attention processes to model global context and capture long-range relationships across spectro-temporal data (27). These capabilities allow ViTs to efficiently identify subtle articulatory irregularities that may span distant temporal and frequency segments, allowing them to outperform CNNs in terms of global context modeling (28). However, standalone ViT architectures have drawbacks in addition to computational complexity, including the need for extensive training datasets and a focus on global attentions, leading to overlooking critical, subtle local markers required for precise disorder differentiation (29).

In light of these shortcomings, hybrid CNN-ViT designs have been used as a potential alternative (30). These hybrid architectures provide superior representational capacity by integrating the local pattern extraction capabilities of CNNs with the global contextual insights of ViTs (31). However, hybrid techniques frequently have inadequate feature fusion algorithms. For instance, the feature fusion approaches, such as concatenation and pooling, treat features uniformly, regardless of their contextual importance or discriminative strength. These fusion procedures negatively impact classification performance and interpretability, which dilute crucial speech signals (32). Thus, advanced fusion methods are required to integrate local and global information of contextual values.

Although these disorders may arise from different neurological, physiological, or functional causes, they often share overlapping acoustic characteristics, making automated identification challenging. Therefore, this study focuses on SD detection under a binary classification setting, where the primary objective is to distinguish healthy individuals speech from individuals with speech impairment rather than to perform fine-grained multi-class categorization of specific disorder subtypes. This scope is adopted to support robust and interpretable detection of abnormal speech patterns across heterogeneous pathological conditions. The major challenge addressed in this study is the limited ability of existing models to jointly capture localized temporal irregularities and long-range contextual dependencies in speech signals. The novel contributions of this study are as follows:

1. Development of an end-to-end, raw waveform diagnostic pipeline using one-dimensional CNN with ViT’s self-attention mechanism.

By leveraging a shallow stack of temporal features, including micro-tremors, jitter, and shimmer without Mel-spectrogram generation. A lightweight ViT’s encoder processes the extracted temporal feature maps, capturing long-range interactions, such as prosodic drift or breadth-to-phenome dependencies. This strategy minimizes the preprocessing latency and builds an end-to-end raw waveform pipeline.

2. Implementation of adaptive local-global fusion.

This study introduces a fusion layer that adaptively re-weights the extracted features. The assignment of specific weights to each feature allows the proposed model to emphasize the diagnostically informative representation for each voice sample. The weights are optimized through back-propagation during the training phase. This adaptive fusion approach yields a compact and highly discriminative fused embeddings, surpassing noise and improving generalization.

3. Deployment of class-attention transformer (CaiT)-based feature refinement.

In this study, CaiT is introduced to refine the global representation, emphasizing salient global features while preserving detailed local features. By employing limited number of CaiT layers, the model achieves better outcomes with minimal computational and memory footprints, enabling the model deployment on real-time or edge-based clinical applications.

4. Enhancement of model explainability through gradient-weighted class activation mapping (Grad-CAM).

In order to foster interpretability, two-tier saliency analysis is implemented. Grad-CAM reveals the events, such as tremor-bursts or articulation breaks using one-dimensional CNN filters with attention-map visualization from the transformer leading to the generation of a diagnostically salient waveform highlighting the model’s prediction.

The remaining part of this study is structured into five key sections, ensuring a logical and coherent flow of ideas and findings. Section 2 offers a comprehensive review of existing approaches in speech pathology detection using spectrogram-based methods, convolutional architectures, and transformer models. The proposed research methodology is described in section 3, outlining data acquisition, preprocessing, feature extraction, fusion, classification, and experimental setup. Section 4 showcases quantitative evaluations across multiple SD and comparisons with baseline models. Section 5 delivers an in-depth discussion of the study outcomes, limitations, and implications for early SD diagnosis. Lastly, section 6 concludes the study through the summarization of major findings and outlines future research directions.

## 2 Related works

In recent years, research on speech disorders has received increased attention, as identifying such disorders can help with early diagnosis of neurological diseases and vocal pathologies (33). Classical algorithms mostly use hand-crafted acoustic features, such as jitter, shimmer, MFCCs, and harmonic-to-noise ratio, combined with traditional machine learning models, such as support vector machine, random forests, and gradient boosting algorithms (34, 35). These methods produce comprehensible results, but they are unable to capture high-dimensional temporal dependencies characteristic of abnormal speech.

Speech convolutional analysis typically uses Mel-spectrograms (36). By approximating cochlear response and compressing high-frequency resolution, the non-linear Mel filterbank produces a two-dimensional representation (36). This representation fits into the CNNs’ spatial inductive biases: two-dimensional kernels record formant trajectories or transient bursts. Accelerating convergence and allowing relatively shallow CNNs to capture the general spectral envelope, reducing pitch-harmonic redundancy, and stabilizing amplitude variation (37, 38). While pathological tremor and dysarthric jitter manifest as phase micro-instabilities, the Mel filterbank excludes phase (38). As a result, CNNs and ViTs are unable to identify the crucial SD patterns. They focus on languid vowels and explosive plosives with equal kernels due to uniform tiling, which blurs transients or underrepresents long-term prosody (39). Static Mel scaling poses significant challenges in SD classifications, hindering augmentation and increasing preprocessing latency throughout the training and deployment stages (39). Additionally, existing hybrid models generally lack mechanisms for intuitive explainability, which is crucial for clinical acceptability (39). Commonly used visualization techniques using CNNs or ViTs may not provide clinically relevant insights (39). While CNN-based methods can neglect to take larger contexts into account, the diffuse global attention maps generated by ViTs make it challenging to identify which auditory regions substantially impact predictions (39).

CNNs encode speech signals with Mel-spectrogram representations, where the perceptual Mel scale is used to scale the frequency axis, and temporal differences are maintained (39). In this form, CNNs use time-frequency bin convolutional kernels to identify localized patterns, including sudden changes in energy, harmonic deviations, and anomalous spectral distributions, which are related to disordered speech (40). These filters gradually learn the low-spectral edges and high-level phonatory structures via hierarchical feature maps. Nonetheless, CNNs have smaller receptive fields, which limit their capability to learn long-range temporal relationships. ViTs solve this problem by splitting the Mel-spectrogram into fixed-sized patches, which are embedded and processed linearly with self-attention mechanisms. This allows the model to estimate pair-wise relationships between remote time-frequency regions and detect globally distributed anomalies across speech segments. Hybrid CNN–ViT models first utilize CNN layers to generate compact feature maps from mel-spectrograms, preserving fine-grained spectral details (40). These features are then re-modeled into token sequences and fed to transformer layers, where self-attention is used to sharpen relationships with global contexts. The combined processing improves the detection of localized spectral anomalies and long-range temporal patterns in speech disorders.

Table 1 summarizes SD detection studies, focusing on how deep learning models extract key features to identify SD at the initial stages. It offers a comparative summary of datasets, input representations, feature extraction strategies, and limitations.

**TABLE 1:** Characteristics of the existing approaches.

Authors	Dataset	Feature extraction	Model	Findings	Limitations
Sait et al. (41)	SVD	MobileNet V3-EfficientB7-Linformer-Performer-based feature extraction	XGBoost with SHAP values	Reported competitive results with an accuracy of 98.90%, precision of 98.00%, recall of 98.10%, F1-score of 98.10%, and specificity of 97.80%, using 12.3 million parameters.	Limited to specific speech disorders; high computational demands of the multi-model ensemble; requires validation on larger and more diverse real-world datasets.
Abdullah et al. (42)	SVD	Filter and Wrapper Methods with K-nearest neighbor	Fully Connected Layer with Sigmoid function	Achieved an accuracy of 95.00% and precision of 98.00%.	Small dataset size; risk of overfitting from optimized feature selection; lack of external/multi-center validation; not tested in noisy real-world environments.
Ribas et al. (43)	SVD	CNN	Random Forest	Obtained an accuracy of 93.90%.	Focused on general voice disorders rather than PD specifically; performance dependency on recording conditions; limited interpretability of self-supervised features.
Hegde et al. (44)	SVD	CNN and ViT	Fully Connected Layer with Sigmoid function	Reported an accuracy of 90.00%, precision of 90.30%, and F1-score of 83.7%.	Study targets Vocal Cord Paralysis (not PD); limited to sustained vowels only; small dataset; pre-trained models may have poor generalization to continuous speech.
Karan et al. (45)	PC-GITA Database	Filter and Wrapper methods	Support Vector Machine (SVM)	Achieved an accuracy of 90.00%.	Small number of participants; computationally intensive Hilbert spectrum analysis; limited feature diversity; requires testing on larger and multi-lingual cohorts.
Rana et al. (46)	PD Dataset	Filter and Wrapper methods	SVM and ANN	Attained an SVM accuracy of 87.00% while the Artificial Neural Network reached 96.7% accuracy.	Relies on a single small dataset limiting generalizability; does not incorporate multimodal data (e.g., gait, handwriting); filter methods may overlook useful features.
Hoq et al. (47)	PD Dataset	Sparse autoencoder	SVM	Achieved an accuracy of 93.5% and F1-score of 95.1%.	Small dataset size; lack of external validation; model may not generalize well across different languages, accents, or recording devices.
Ali et al. (48)	PD Dataset	Deep neural network-based feature extraction	SVM	Reported an accuracy of 95.00%, sensitivity of 100.00%, and specificity of 90.00%.	Missing disease severity and medication state (ON/OFF) information; did not address differential diagnosis between idiopathic and atypical Parkinsonism; binary classification has limited clinical significance.
Faragó et al. (49)	27 participants (Private Dataset)	Speech spectrograms, speech energy spectrograms, Mel spectrograms; Wiener filtering for noise reduction; segments fed directly as images to CNN	MobileNet CNN	MobileNet achieved an outstanding accuracy of 96% on speech energy spectrograms after Wiener filtering and dataset reduction; raw speech spectrograms reached 93%, while Mel spectrograms attained 92%; Wiener filtering consistently improved overall performance by 8–12%.	Small dataset; short voiced segments limit tremor/formant analysis; Wiener filter less effective on complex/non-stationary noise
Rahmatallah et al. (50)	PD	Linear-scale and Mel-scale spectrogram images (1.5 s segments); pre-trained CNN with transfer learning (Inception V3)	Inception V3	Inception V3 achieved excellent AUC scores of 0.95–0.97 on the UAMS dataset and 0.92–0.95 on the mPower dataset; Mel-scale spectrograms were consistently superior (*p* < 0.001) and the model significantly outperformed traditional ML approaches based on acoustic/spectral features.	Self-reported diagnoses in mPower (potential mislabeling); small UAMS cohort; limited interpretability of which spectrogram patterns drive CNN decisions
Jeong et al. (51)	Custom Korean dataset	Log Mel filterbank spectrograms (128-bin, SpecAugment + Mixup augmentation); input to Audio Spectrogram Transformer (AST) and PSLA (CNN-based) models	CNN	The CNN model achieved a strong accuracy of 92.15%, sensitivity of 91.53%, specificity of 92.79%, and AUC of 97.43% and Grad-CAM confirmed the model focused on PD-specific high-frequency muffling patterns.	Small dataset and single-language (Korean) limits generalizability; no per-task analysis or fusion; potential overfitting in transformer model
Chen et al. (52)	Private Dataset	STFT spectrograms multi-model feature fusion of pre-trained CNNs (DenseNet121, MobileNetV3-Large, ShuffleNetV2) via summation	Multi-model fusion (MobileNetV3 + ShuffleNetV2)	MobileNetV3 + ShuffleNetV2 delivered a high accuracy of 95.56%, AUC of 0.99, and F1-score of 95.47%; all fusion models outperformed single CNNs, and the approach was successfully validated on the public Figshare dataset (∼92% accuracy).	Single-center, single-language, single-text task; limited diversity in age/dialects; needs larger multi-lingual datasets
Quamar et al. (53)	PD	Mel-spectrograms, MFCCs, raw waveforms, plus acoustic features	Bidirectional Long Short-Term Memory (BiLSTM)	The BiLSTM model achieved the highest accuracy of 97%, AUC of 0.95, and F1-score of 0.97; CNN+GRU reached 94% and CNN+LSTM 91%; all deep learning models vastly outperformed traditional ML baselines (44–55% accuracy).	Small dataset size; low-quality telephone recordings with high variability; no external/clinical validation; traditional ML sensitive to scaling & feature selection

Although there have been dramatic improvements in detecting speech disorders, there are still some crucial shortcomings. Mel-spectrogram-based CNN models are effective at capturing localized time-frequency variations but do not capture long-range temporal variations in speech sequences. Methods based on transformers enhance global contextual modeling but tend to lose fine-grained spectral details due to patch-wise tokenization. Current hybrid CNN-ViT models strive to harness the advantages of both, but they typically use fixed fusion processes, which fail to dynamically weigh feature inputs across channels. Moreover, the conversion of fused representations into structured tokens is not adequately defined, which limits reproducibility. Subject-level data splitting and external validation are also lacking in many studies, leading to possible data leakage and overestimation of performance. Furthermore, there has been little focus on interpretable localization of disorder-relevant acoustic regions. These limitations drive the framework suggested, which incorporates channel-wise adaptive fusion, CaiT-based feature refinement, subject-independent evaluation, and attention-guided interpretability to detect SD.

## 3 Materials and methods

**FIGURE 1:**
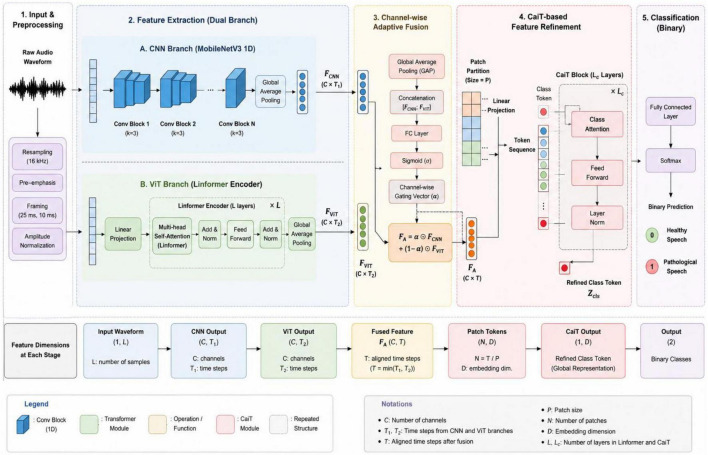
Proposed binary SD approach using CaiT-based feature refinement.

The proposed methodology leverages a structured pipeline integrating the strength of one-dimensional CNN, ViT, adaptive feature fusion, and CaiT architectures. Figure 1 depicts the proposed methodology for identifying SD using the raw speech waveforms. Unlike traditional approaches rely on spectrograms, the proposed approach processes raw speech waveforms through convolutional layers, capturing acoustic anomalies, including tremors, jitter, and shimmer, associated with speech impairments. The temporal feature maps are divided into patches of fixed length. These patches are linearly projected to produce embeddings for transformer processing. With ViT’s self-attention layers and positional embeddings, the model contextualizes these patches and capture PD’s prosodic and rhythmic disruptions. The adaptive fusion process dynamically assigns weights to each stream depending on their relative relevance, mitigating redundancy and improving the discriminative quality of the features. Subsequently, CaiT blocks are introduced into the pipeline after fusion, refining the combined feature representation.

Through the selective aggregation of diagnostic indicators and the reduction of computational complexity, this additional refinement step guarantees the production of a meaningful global summary. By using this enhanced embedding using a lightweight, fully-connected layer with a softmax layer, reliable SD classification. By leveraging Grad-CAM and attention-based visualizations, the proposed model provides dual-layered interpretability, visualizing local anomalies and long-range acoustic patterns responsible for the model’s decisions.

### 3.1 Data acquisition

Three public datasets were used to train and evaluate the proposed model. Saarbruecken voice database (SVD) (54) contains 2,043 voice recordings. It covers multiple disorders, including vocal fold nodules, paralysis, laryngitis, and Parkinson’s-related dysarthria. The SVD dataset as the primary dataset for model training and internal evaluation. In this study, all pathological voice samples are grouped under the SD class, while normal voice recordings are treated as the healthy class. The model learns discriminative temporal and acoustic patterns from raw speech waveforms to distinguish SD individuals from healthy controls. Subject-level splitting is applied to prevent speaker leakage and ensure unbiased evaluation.

In order to maintain subtle vocal attributes, such as jitter, shimmer, and harmonic-to-noise ratios, recordings are sampled at a high fidelity (typically 50 kHz, 16-bit PCM). The glottal vibration patterns based on EGG signals are complementary to the acoustic waveforms, facilitating multimodal analysis. The detailed sustained-vowel recordings support the development of deep learning-based voice pathology detection. Parkinson’s speech database (55) includes voice recordings of 20 PD patients and 20 healthy individuals. It covers multiple types of voice recordings (sustained vowels, numbers, words, and short sentences). An expert physician evaluates each patient using the unified PD rating scale.

An additional external dataset, the VOICED dataset (56), was used to further evaluate the generalization capability of the proposed model. VOICED is a publicly available pathological voice dataset containing 208 clinically verified voice samples, including 150 pathological voices and 58 healthy voices from adults aged 18–70 years. The dataset includes sustained vowel /a/ recordings along with clinical and demographic information such as age, gender, pathology, Voice Handicap Index, and Reflux Symptom Index. Since VOICED is independent from the SVD and PD datasets and includes heterogeneous pathological voice conditions, it provides a suitable benchmark for assessing cross-dataset robustness.

In order to conduct an unbiased analysis, all splitting procedures were performed at the subject level, not the recording level or the segment level. Using this strategy, there is no overlap of any kind between the training and test sets, since samples from the same subjects cannot appear in both sets. Model training and optimization were performed using the SVD dataset, which was split into training and internal test sets using a subject-wise split. The PD dataset was used solely for external validation purposes. No samples from the PD dataset were used during the training and internal evaluation phases. In addition, the augmentation strategy was applied only to the training set, and the internal test and external validation sets remained unchanged throughout the experiment. The crucial features of the datasets are discussed in Table 2.

**TABLE 2:** Key features of datasets.

Dataset	Purpose	Total samples/ subjects	Recording type	Evaluation protocol	Relevance to the study
SVD dataset	Training and internal testing	2,043 voice recordings	Sustained vowel recordings and pathological voice samples	Subject-level split for internal validation	Used as the primary dataset to train and evaluate binary pathological speech detection
PD dataset	External validation	40 subjects: 20 PD and 20 healthy	Sustained vowels, words, numbers, and short sentences	Used only for external validation; no training samples included	Evaluates the model’s generalization to unseen neurological speech data
VOICED dataset	Additional external validation	208 voice samples: 150 pathological and 58 healthy	Sustained vowel /a/ recordings	Used only for additional external validation	Assesses robustness across heterogeneous pathological voice conditions

### 3.2 Data preprocess and augmentation

Although SD may include different pathological subtypes, the present experimental design does not aim to perform fine-grained multi-class disorder classification. Instead, the pathological speech samples are treated as a single positive class, while healthy speech samples are treated as the negative class. This binary formulation enables the model to learn generalized acoustic and temporal abnormalities associated with SD while maintaining a reliable and reproducible evaluation protocol.

We employ preprocessing and augmentation techniques to optimize CNNs and ViTs architecture performance. The preprocessing strategy is essential to ensure model training and generalization. Uniform resampling is crucial in capturing consistent temporal resolution across audio waveforms. It allows convolutional kernels to detect micro-temporal acoustic features, including tremor and jitter. Amplitude normalization is applied to scale waveforms within a defined range of Flanagan et al. (1), mitigating differences emerging from diverse microphone sensitivities, speaker volume, and recording environment. In addition, a segmentation strategy is used to segment the audio signals into fixed-length segments, providing consistent input dimensions and clearly defined patch boundaries.

Data augmentation techniques, including pitch shifting, time stretching, amplitude scaling, and additive Gaussian noise, are used to enhance the model’s robustness and generalization. Pitch shifting and time stretching simulate natural voice variability and speaking rate differences. These techniques improve the model’s resilience to speakers’ variability. Gaussian noise injection and amplitude scaling enable the model to handle real-world acoustic interference.

### 3.3. Feature extraction

**FIGURE 2:**
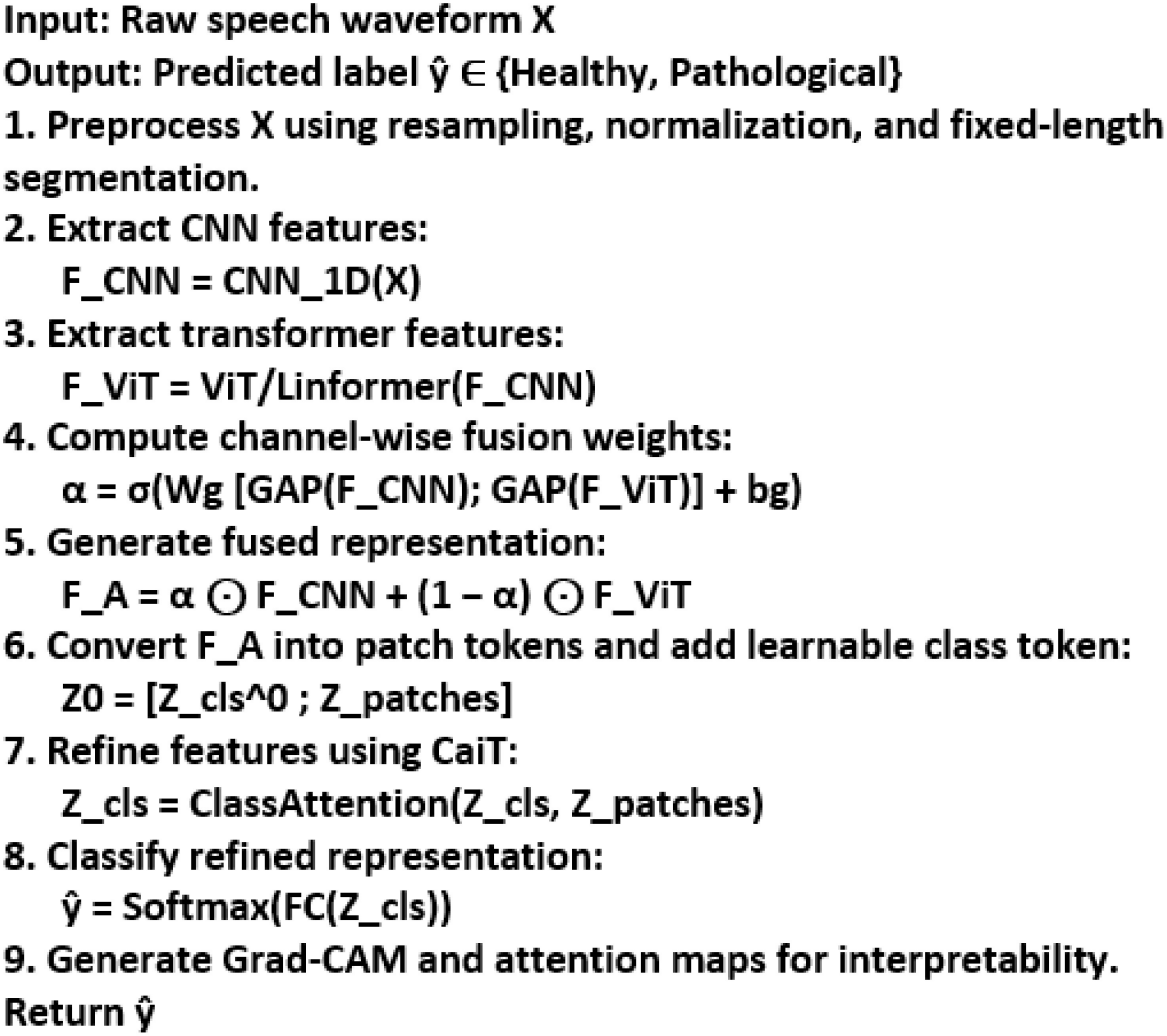
Pseudocode of the proposed binary SD detection.

To extract meaningful representations from raw audio waveforms, a hybrid architecture combining a 1D CNN and a ViT is employed. This hybrid approach leverages the potential of CNNs in capturing fine-grained temporal features and the global context modeling capabilities of transformers. Figure 2 summarizes the complete workflow of the proposed binary pathological speech detection framework. Initially, the model extracts local temporal features using a one-dimensional CNN and global contextual features using the ViT/Linformer encoder. These representations are integrated via channel-wise adaptive fusion, refined with CaiT-based class attention, and finally classified as pathological or healthy speech using a fully connected softmax layer.

Using a stack of convolutional layers, the raw audio waveform is divided into overlapping temporal segments. MobileNet V3 is a lightweight, efficient convolutional neural network suitable for edge computing environments. The convolutional blocks are initialized using MobileNet V3 weights. The mathematical form of the convolutional operation is outlined in Equation 1.


$$F_{C\,N\,N}\,\left(t\right)=\sigma \,\left(\sum_{k=1}^{K}x_{k}\,\left(t\right)*w_{i\,k}\,\left(t\right)+b_{i}\right)$$ (1)

where *F*_*CNN*_(*t*) is the output feature map, *x*_*k*_(*t*) represents the input waveform segment at channel *K*, *w*_*ik*_(*t*) indicates convolutional kernels, *b_i_* is the bias term, and σ is the non-linear activation function.

The convolutional operation identifies crucial patterns, including frequency shifts, silence intervals, and pitch modulation, associated with neurological and psychological conditions. Subsequently, a transformer encoder is used to process the high-level features, extracting features (*F*_*ViT*_), including patterns of speech or repetition related to cognitive disorders. The CNN features are extracted using the convolutional layers. The final set of convolutional layers is frozen and integrated with the Linformer’s self-attention mechanism. A global average pooling layer is used to flatten the features in order to support the Linformer’s architecture. By projecting the key and value matrices into a lower-dimensional space, Linformer’s self-attention reduces complexity. This dual approach detects micro-level acoustic cues and macro-level speech patterns, providing valuable diagnostic information. It learns robust representations without substantial computational overhead, making it suitable for early SD diagnosis.

### 3.4 Adaptive feature fusion

Concatenation of the extracted features often resulting in suboptimal performance due to the incorporation of irrelevant information. To address this limitation, a channel-wise adaptive feature fusion mechanism is proposed that integrates complementary representations from the CNN and ViT branches in a context-aware manner. Through a channel-wise gating vector, fine-grained control over feature contributions is enabled. Let *F*_*CNN*_, *F*_*ViT*_ ∈ ℝ^*C*×*T*^ represent feature maps where *C* denotes the number of channels and *T* represents the temporal dimension. Equation 2 computes the fused feature representations.


$$F_{A}=\propto ⊙F_{C\,N\,N}+\left(1-\propto \right)⊙F_{V\,i\,T}$$ (2)

where *F_A_* is the fused feature, ∝ is a channel-wise adaptive gating vector, and⊙ denotes channel-wise broadcast multiplication. Equation 3 computes the channel-wise gating vector that learns dynamically using global contextual information from feature streams. Initially, global average pooling (GAP) captures channel-wise statistics, followed by a learnable transformation.


$$\propto =\sigma \left(W_{g}\left[GAP\left(F_{C\,N\,N}\right);GAP\left(F_{V\,i\,T}\right)\right]+b_{g}\right)$$ (3)

where *W*_*g*_ and *b*_*g*_ denote the weight matrix and bias term of the gating fusion, respectively, “;″ represents the feature concatenation and σ(.) is the sigmoid activation function.

With this framework, independent weights are assigned to each channel, leading to a more descriptive and discriminative approach to feature fusion in contrast to scalar gating. The proposed scheme thus successfully emphasizes informative patterns while filtering out noise. Additionally, channel-level feature fusion enables greater collaboration between local patterns learned by CNNs and global context learned by transformer models. This leads to improved accuracy and better generalization.

### 3.5 CaiT-based feature refinement

In order to refine the fused features, a CaiT module is introduced. The fused feature representation (*F*_*A*_) is transformed into a sequence of tokens prior to entering the CaiT block. Initially, the temporal dimension *T* is partitioned into *N* non-overlapping patches of equal size *P*, such that $N=\frac{T}{P}$, capturing the local temporal information. Each patch is transformed into a vector dimension *C*×*P*.Subsequently, these patch vectors are projected into a *D*-dimensional space by leveraging a learnable linear projection function as shown in equation 4.


$$Z_{p\,a\,t\,c\,h\,e\,s}=P\,r\,o\,j\,\left(F_{A}\right)\in {\mathbb{R}}^{N\times D}$$ (4)

where *Z*_*patches*_ denotes the sequence of embedded patch tokens, *D* is the embedding dimension, and *Proj*(.) Indicates a linear transformation applied independently to each patch. A learnable class token $Z_{c\,l\,s}^{0}\in {\mathbb{R}}^{1\times D}$ is initialized and included in the patch token sequence, incorporating global contextual information. Equation 5 outlines the process of passing input to the CaiT block.


$$Z_{0}=\left[Z_{c\,l\,s}^{0};Z_{p\,a\,t\,c\,h\,e\,s}\right]\in {\mathbb{R}}^{\left(N+1\right)\times D}$$ (5)

where *Z_0_*denotes the initial token sequence consisting of one class token and *N* patch tokens.

Furthermore, a class-attention mechanism is used to refine features within the CaiT architecture. Using this mechanism, the class token interacts with the patch tokens using attention operations. Equation 6 shows the updating procedure of the class token at the layer (*l*).


$$Z_{c\,l\,s}^{l}=C\,l\,a\,s\,s-a\,t\,t\,e\,n\,t\,i\,o\,n\,\left(Z_{c\,l\,s}^{l-1},Z_{p\,a\,t\,c\,h\,e\,s}\right)$$ (6)

where $Z_{c\,l\,s}^{l-1}$ is the class token from the previous layer and *Z*_*patches*_ remain fixed as the set of patch tokens. The class-attention approach helps the class token extract information from all patch tokens that relate to the important features of interest. The iterative process extracts diagnostic features such as temporal irregularities, pauses, and tonal variations while ignoring irrelevant features. The refined class tokens (*Z*_*cls*_) serve as the input to the subsequent classification layer for final prediction, contributing to model interpretability.

### 3.6 Interpretable feature classification

Using Grad-CAM and attention-based visualization with the final classification layer, we enable the model’s interpretability. Visual heatmaps are generated by integrating Grad-CAM into the CNN layers, highlighting the time segments that influence the model’s prediction. Equation 7 presents the mathematical form of the Grad-CAM visualization.


$${ℒ}_{G\,C}=R\,e\,L\,u\,\left(\sum_{k}\alpha_{k}^{C}\,A^{k}\right)$$ (7)

where *ℒ_GC_* is the heatmap, *A^k^* is the activation map from the last convolutional layer, $\alpha_{k}^{C}$ is the importance weights obtained through global average pooling of the gradients of the target class (*C*), and *ReLu* is the rectified linear function to indicate the significant part of the waveform.

Likewise, we visualize attention scores from the transformer layer using the attention matrix, uncovering long-range dependencies in the audio signal. Equation 8 shows the computation of the attention matrix.


$$A\,\left(Q,K\right)=S\,o\,f\,t\,m\,a\,x\,\left(\frac{Q\,K^{T}}{\sqrt{d_{k}}}\right)$$ (8)

where *A*(*Q*,*K*) is the attention matrix with query (*Q*) and key (*k*), *QK^T^* is the dot product between the query and key values, $\frac{1}{\sqrt{d_{k}}}$ is the scaling function, and *Softmax* function transforms the scaled dot products into a probability distribution over the keys.

This dual interpretability approach provides valuable insights, allowing clinicians and voice experts to make effective decisions. To optimize the proposed model’s performance, BOHB is employed. BOHB combines the exploration strengths of Bayesian optimization with Hyperband. Using BOHB, hyperparameters, including learning rate, batch size, depth of CNN and ViT, and embedding dimensions, are optimized, achieving high accuracy while maintaining computational efficiency.

To strengthen the interpretability assessment, the Grad-CAM and attention-based visualization outputs were evaluated both quantitatively and clinically. First, the highlighted temporal regions generated by Grad-CAM and transformer attention maps were compared with abnormal acoustic regions identified from waveform patterns. The agreement between model-highlighted regions and clinically relevant temporal segments was assessed using localization overlap and expert agreement analysis. Clinical experts/speech-language specialists reviewed the highlighted waveform regions and evaluated whether they corresponded to meaningful pathological speech characteristics, including unstable phonation, abnormal pauses, amplitude irregularity, pitch instability, tremor-like fluctuations, and articulation disruption. This validation was performed to determine whether the model explanations were consistent with clinically recognizable speech abnormalities. In addition, the highlighted regions were compared with known acoustic indicators of disordered speech to assess whether the model focused on diagnostically meaningful speech segments rather than irrelevant background or silent regions.

### 3.7 Performance evaluation

A comprehensive set of metrics: accuracy, precision, recall, F1-score, specificity, and confidence intervals, is used to determine the model’s ability to correctly identify PD patients and healthy individuals. The statistical validation of the model performance is critical for the demonstration of its reliability and reproducibility in the medical field (57–59). In the realm of statistical tests, the McNemar’s test is especially important for assessing classification models, as it allows determining whether there are statistically significant differences between outcomes from two models or whether the difference is simply accidental. Furthermore, the standard deviation provides information on the consistency of model performance across different samples. The narrower the interval of dispersion, the more stable the model’s behavior. The confidence interval (CI) helps improve the reliability of experimental results, ensuring that the accuracy is based on an adequate statistical analysis and can be interpreted correctly. Root Mean Square Error (RMSE) is another important metric that represents the difference between predicted and observed output values (60–62). Equations (9)–(13) outline the core evaluation metrics, including accuracy, precision, recall, F1-score, and specificity, while Equations (14)–(16) define RMSE, standard deviation, and confidence interval. Equation (17) presents McNemar’s test, which collectively ensures a comprehensive assessment of classification performance, prediction error, stability, reliability, and statistical significance.

To ensure a comprehensive and statistically grounded evaluation, multiple performance metrics were employed. Let True Positive (TP), True Negative (TN), False Positive (FP), and False Negative (FN) denote the classification outcomes.

The Accuracy measures overall correctness:


$$\mathrm{Accuracy}=\frac{T\,P+T\,N}{T\,P+T\,N+F\,P+F\,N}$$ (9)

The Precision quantifies the proportion of correctly predicted positive cases:


$$\mathrm{Precision}=\frac{T\,P}{T\,P+F\,P}$$ (10)

The Recall (Sensitivity) evaluates the ability to detect actual positive cases:


$$\mathrm{Recall}=\frac{T\,P}{T\,P+F\,N}$$ (11)

The F1-score, a harmonic mean of precision and recall, is defined as:


$$\mathrm{F1}-\mathrm{score}=\frac{2\cdot \left(P\,r\,e\,c\,i\,s\,i\,o\,n\cdot R\,e\,c\,a\,l\,l\right)}{P\,r\,e\,c\,i\,s\,i\,o\,n+R\,e\,c\,a\,l\,l}$$ (12)

The Specificity measures the correct identification of negative cases:


$$\mathrm{Specificity}=\frac{T\,N}{T\,N+F\,P}$$ (13)

To quantify prediction error, RMSE is computed as:


$$\mathrm{RMSE}=\sqrt{\frac{1}{N}\,\sum_{i=1}^{N}{\left(y_{i}-{\hat{y}}_{i}\right)}^{2}}$$ (14)

where *y_i_*and ${\hat{y}}_{i}$represent actual and predicted labels, respectively.

For statistical reliability, the Standard Deviation (SD) is calculated as:


$$\sigma =\sqrt{\frac{1}{N}\,\sum_{i=1}^{N}{\left(x_{i}-\mu \right)}^{2}}$$ (15)

The 95% Confidence Interval (CI) is estimated as:


$$C\,I=\mu \pm 1.96\cdot \frac{\sigma }{\sqrt{N}}$$ (16)

Finally, McNemar’s test is used to assess statistical significance between paired classifiers:


$$\chi^{2}=\frac{{\left(|b-c|-1\right)}^{2}}{b+c}$$ (17)

where *b*and *c*represent the number of discordant predictions between two models.

## 4 Results

To ensure an in-depth evaluation of the proposed SD detection model, the experimental settings are carefully designed. The model performance is assessed in terms of the classification performance and computational efficiency. Utilizing an NVIDIA RTX A6000 GPU with 48 GB of RAM and the CUDA 11.6 environment, the experiments were conducted in the PyTorch deep learning framework. The computational environment is selected to meet the high memory and computational demands of transformer-based designs for comprehensive hyperparameter identification. To provide an unbiased and statistically relevant assessment, the SVD dataset (29) is split into training (70%), validation (15%), and test (15%) sets, maintaining the class distribution across subsets. Additionally, the model is generalized on Parkinson’s speech database (30). To enable reproducibility, Table 3 describes the complete architecture of layers, feature dimensionality, tokenization settings, attention mechanism, normalization technique, loss function, optimization process, and the approach for calculating the computational complexity of the proposed system. The architecture consists of raw waveform input data processing by the one-dimensional CNN branch, a contextual encoder using transformers, a channel-adaptive fusion layer, a CaiT-based refinement block, and a binary classification head.

**TABLE 3:** Experimental configuration of the proposed model.

Category	Component/Layer	Configuration/search range/selected value	Output/feature dimension
Input	Raw audio waveform	Sampling rate = 16 kHz; segment length = 3 s	1 48,000
Preprocessing	Amplitude normalization	Waveform normalized to (1)	–
	Segmentation	Fixed-length waveform segmentation; augmentation applied only to training set	
CNN feature extractor	CNN layer search space	BOHB search range: 3-5 convolutional layers; selected value = 4 layers	
	Conv1D Block 1	Conv1D 1 - > 32, kernel size = 11, BatchNorm, ReLU, MaxPool1D s = 2, Dropout = 0.3	32 24,000
	Conv1D Block 2	Conv1D 32 - > 64, kernel size = 11, BatchNorm, ReLU, MaxPool1D s = 2, Dropout = 0.3	64 12,000
	Conv1D Block 3	Conv1D 64 - > 128, kernel size = 11, BatchNorm, ReLU, MaxPool1D s = 2, Dropout = 0.3	128 6,000
	Conv1D Block 4	Conv1D 128 - > 128, kernel size = 11, BatchNorm, ReLU, MaxPool1D s = 2, Dropout = 0.3	128 3,000
Token generation	Patch formation	Non-overlapping temporal patches; patch size P = 16	N = T/P
	Linear projection	Patch vectors projected into embedding space	N × D
Transformer encoder	Transformer type	ViT with Linformer self-attention	–
	Transformer depth	BOHB search range: 2-4 layers; selected value = 3 layers	N× 256
	Attention heads	4 attention heads	N 256
	Embedding dimension	BOHB search range: 128-256; selected value = 256	D = 256
	Normalization and activation	LayerNorm and GELU activation	N × 256
Transformer output	Global contextual features	Transformer features projected to CNN-compatible channel dimension	128 × T
Adaptive fusion	Channel-wise fusion gate	GAP applied to F_CNN and F_ViT; concatenation, FC layer, sigmoid activation	Alpha in *R*^128^
Adaptive fusion	Fused feature representation	F_A = alpha * F_CNN + (1-alpha) * F_ViT	128 × T
CaiT refinement	Patch decomposition	F_A decomposed into patch tokens and one learnable class token	(N+1) × 256
	CaiT layers	2 class-attention layers	1 × 256
	Attention heads	4 attention heads	1 × 256
	Normalization	LayerNorm after class-attention operation	1 × 256
Classification head	Fully connected classifier	Dropout = 0.3; fully connected layer followed by Softmax	2 classes
Loss function	Classification loss	Cross-entropy loss	Binary pathological/healthy prediction
Optimization	Optimizer	Adam optimizer	–
	Initial learning rate	1 10^−4^	–
BOHB search space	Learning rate	1 10^−5^to 1 10^−3^	–
	Batch size	Search range: 32–64; selected value = 32	–
Optimization	Weight decay	1 10^−4^	–
	Maximum epochs	100 epochs	–
	Early stopping	Patience = 15 epochs	–
Validation protocol	Data splitting	Subject-independent split; augmentation applied only to training data	–
External validation	Independent dataset	External dataset used only for validation, not training or tuning	–
Complexity analysis	Parameters	Counted from all trainable layers	5.2 million
	FLOPs	Estimated using one forward pass with input size 1 × 48,000	3.8 GFLOPs
	Inference time	Average inference time per sample on test data	18.5 ms/sample

BOHB was used to optimize the learning rate, batch size, number of CNNs layers, transformer depth, and embedding dimension. The table reports both the search ranges and the final selected configuration to improve reproducibility.

**FIGURE 3:**
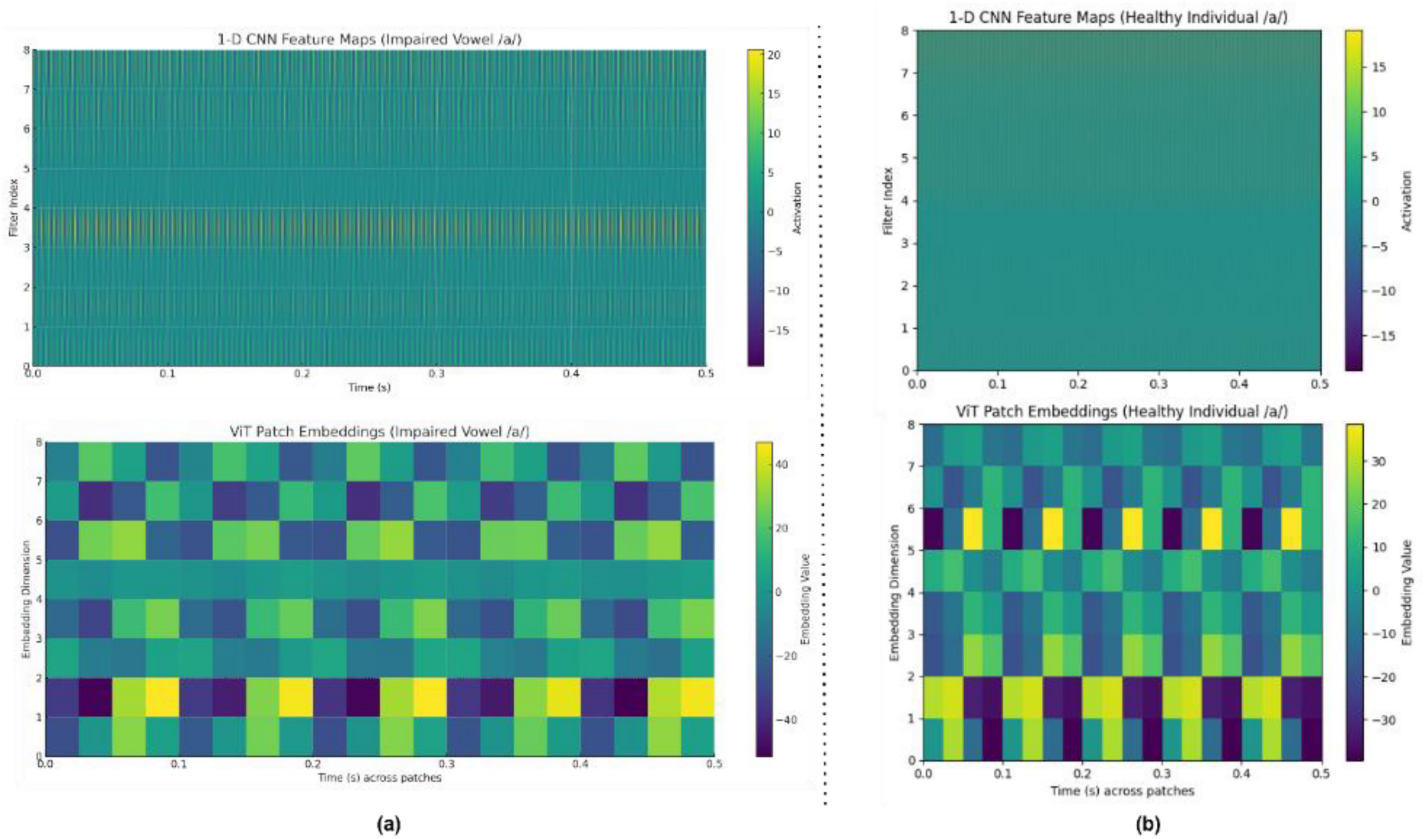
Sample CNN and ViT features of **(a)** speech-impaired and **(b)** healthy individuals.

Figure 3 presents the unique behavior of the proposed model in distinguishing between speech-impaired and healthy individuals using audio waveforms. Figure 3a shows that the appearance of the one-dimensional CNN feature map is uniform, indicating that the temporal features lack sufficient discriminative information. However, ViT patch embedding shows variability, reflecting ViT’s ability in detecting global dependencies. Similarly, Figure 3b presents CNN and ViT feature representations for a healthy individual, exhibiting richer, more structured activation patterns.

**FIGURE 4:**
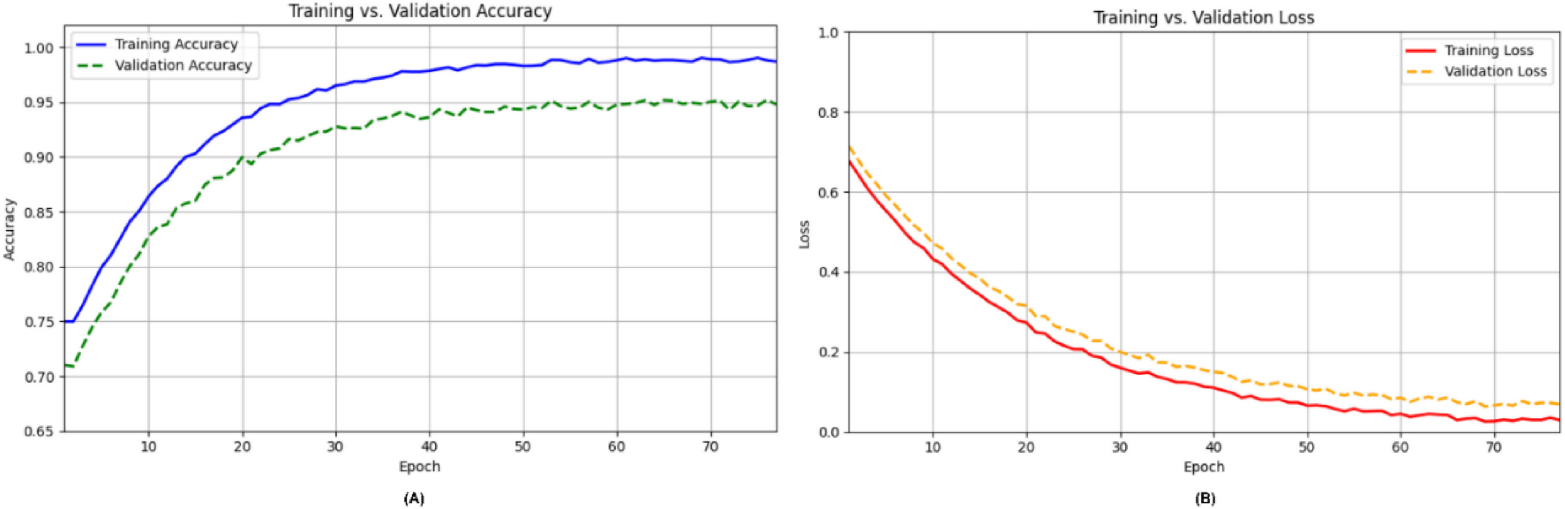
Performance of the proposed SD detection. **(A)** Training and validation accuracy. **(B)** Training and validation loss.

The training and validation performance of the proposed model is depicted in Figure 4. Figure 4A demonstrates that both training and validation accuracy grow continuously in the first epochs, after which their growth rates gradually balance. The training accuracy approaches 98–99%, whereas the validation accuracy reaches and maintains a high level of 94–95%, indicating strong generalization performance. The intermediate distance between the two curves is indicative of effective learning without serious overfitting. Figure 4B also shows the training and validation loss curves, which exhibit a continuous downward trend and converge at Epoch 50–60. The validation loss closely follows the training loss with minor fluctuations, further confirming stable optimization. The plateau observed in subsequent epochs indicates that the model has attained an optimal learning condition, suggesting convergence and robustness to unknown data. The source code for reproducing the study is specified in the Supplementary material.

Table 4 presents the ablation and robustness analysis of the proposed framework. The CNN-only model achieved 89.85% accuracy, indicating that localized temporal feature extraction alone is insufficient for modeling complex pathological speech patterns. The ViT/Linformer-only configuration improved accuracy to 91.20%, reflecting the benefit of global contextual modeling, but its performance remained limited due to weak sensitivity to fine-grained temporal variations. Combining CNN and ViT without fusion increased accuracy to 92.75%, while static fusion achieved 93.80%, demonstrating that local and global features are complementary. Replacing static fusion with channel-wise adaptive fusion improved accuracy to 95.45%, confirming the importance of dynamic feature weighting. The full proposed model achieved the highest accuracy of 97.50% and the lowest RMSE of 0.158, showing that CaiT-based refinement provides a meaningful additional gain by selectively aggregating discriminative patch-level features. Corrected statistical testing confirms that these improvements are significant.

**TABLE 4:** Ablation and robustness analysis using repeated subject-wise validation (SVD dataset).

Model configuration	Accuracy (%)	Precision (%)	Recall (%)	F1-score (%)	Specificity (%)	RMSE	Repeated subject-wise CV mean ± Std (%)	Bootstrap 95% CI	Corrected *p*-value
CNN Only	89.85	88.60	89.20	88.90	88.40	0.318	89.85 ± 3.10	86.90–92.75	0.004
ViT/Linformer only	91.20	90.35	90.80	90.57	90.10	0.297	91.20 ± 2.85	88.55–93.82	0.007
CNN + ViT without fusion	92.75	91.90	92.35	92.12	91.70	0.269	92.75 ± 2.60	90.28–95.10	0.012
CNN + ViT + static fusion	93.80	92.95	93.40	93.17	92.80	0.249	93.80 ± 2.35	91.55–96.02	0.019
CNN + ViT + Channel-wise fusion without CaiT	95.45	94.60	95.20	94.90	94.30	0.213	95.45 ± 2.05	93.40–97.30	0.036
Proposed model: CNN + ViT + channel-wise fusion + CaiT	97.50	97.03	98.00	97.51	97.00	0.158	97.50 ± 1.85	95.80–99.20	—

Table 5 compares the proposed model with the state-of-the-art CNN and transformer-based frameworks on the SVD dataset. The proposed model achieves 97.50% accuracy and balanced performance across precision, recall, F1-score, and specificity, indicating its usefulness for speech-based disorder classification. The proposed approach achieves higher or more stable performance than standalone architectures, including MobileNetV3, RegNetX, EfficientNet-B7, Linformer, and ViT. The improvement observed could be explained by the combination of channel-wise adaptive fusion and CaiT-based feature refinement, which are effective at integrating local temporal representations provided by CNNs with global contextual dependencies provided by transformer architectures. Conversely, CNN-based models are dominated by local patterns but fail to capture long-range dependencies, whereas transformer-based ones can fail to capture fine-grained temporal variations. Notably, statistical validation using the McNemar test shows that the performance gains of the proposed model over baseline approaches are significant (*p* < 0.05). This is a confirmation that the observed gains are not due to random variation but an actual increase in classification ability. Moreover, the comparatively small confidence intervals imply that model behavior is consistent across samples, thereby strengthening the robustness and reliability of the offered method in real-world applications.

**TABLE 5:** Comparison of classification performance: proposed model vs. pre-trained CNN and ViT models (SVD dataset).

Classifier	Accuracy (%)	Precision (%)	Recall (%)	F1-score (%)	Specificity (%)	RMSE	Mean ± Std (%)	95% CI (Accuracy)	McNemar’s *p*-value
Proposed model	97.50	97.03	98.00	97.51	97.00	0.158	97.50 ± 1.85	95.80–99.20	–
MobileNet V3	94.60	92.85	91.70	92.27	92.40	0.233	94.60 ± 2.45	92.20–97.00	0.017
RegNetX	94.95	93.20	92.30	92.75	92.85	0.224	94.95 ± 2.30	92.70–97.20	0.023
EfficientNet B7	94.80	93.40	92.60	93.00	92.90	0.228	94.80 ± 2.10	92.74–96.86	0.038
Linformer	94.55	92.95	92.10	92.52	92.60	0.234	94.55 ± 2.05	92.54–96.56	0.044
ViT	94.20	92.60	91.85	92.22	92.30	0.240	94.20 ± 2.20	92.04–96.36	0.029

Table 6 provides an evaluation of the proposed system on the PD dataset considered as an external independent test set. The model shows uniform prediction capability, yielding an accuracy of 97.50% with high recall and balanced precision values, suggesting that the model can recognize pathological voice patterns well for unseen patients. Unlike previous models that witness reduced performance when tested under cross-dataset scenarios, the proposed system performs well across all evaluation metrics. The reason for such stability stems from its ability to maintain both temporal continuity and context among features during their extraction process. The low RMSE value suggests small prediction errors, and the tight confidence interval implies uniformity of prediction across different data instances.

**TABLE 6:** Comparison of classification performance: proposed model vs. pre-trained CNN and ViT models (PD dataset).

Classifier	Accuracy (%)	Precision (%)	Recall (%)	F1-score (%)	Specificity (%)	RMSE	Mean ± Std (%)	95% CI (Accuracy)	McNemar’s *p*-value
Proposed model	97.50	95.24	100.00	97.56	95.00	0.158	97.50 ± 1.90	95.65–99.35	—
MobileNet V3	92.50	90.10	89.50	89.80	90.20	0.274	92.50 ± 2.80	89.75–95.25	0.014
RegNetX	93.25	91.20	90.40	90.80	90.90	0.259	93.25 ± 2.65	90.65–95.85	0.019
EfficientNet B7	93.80	91.95	91.10	91.52	91.50	0.249	93.80 ± 2.40	91.45–96.15	0.027
Linformer	93.45	91.60	90.75	91.17	91.20	0.255	93.45 ± 2.35	91.15–95.75	0.033
ViT	93.10	91.30	90.20	90.75	90.80	0.263	93.10 ± 2.50	90.65–95.55	0.022

**FIGURE 5:**
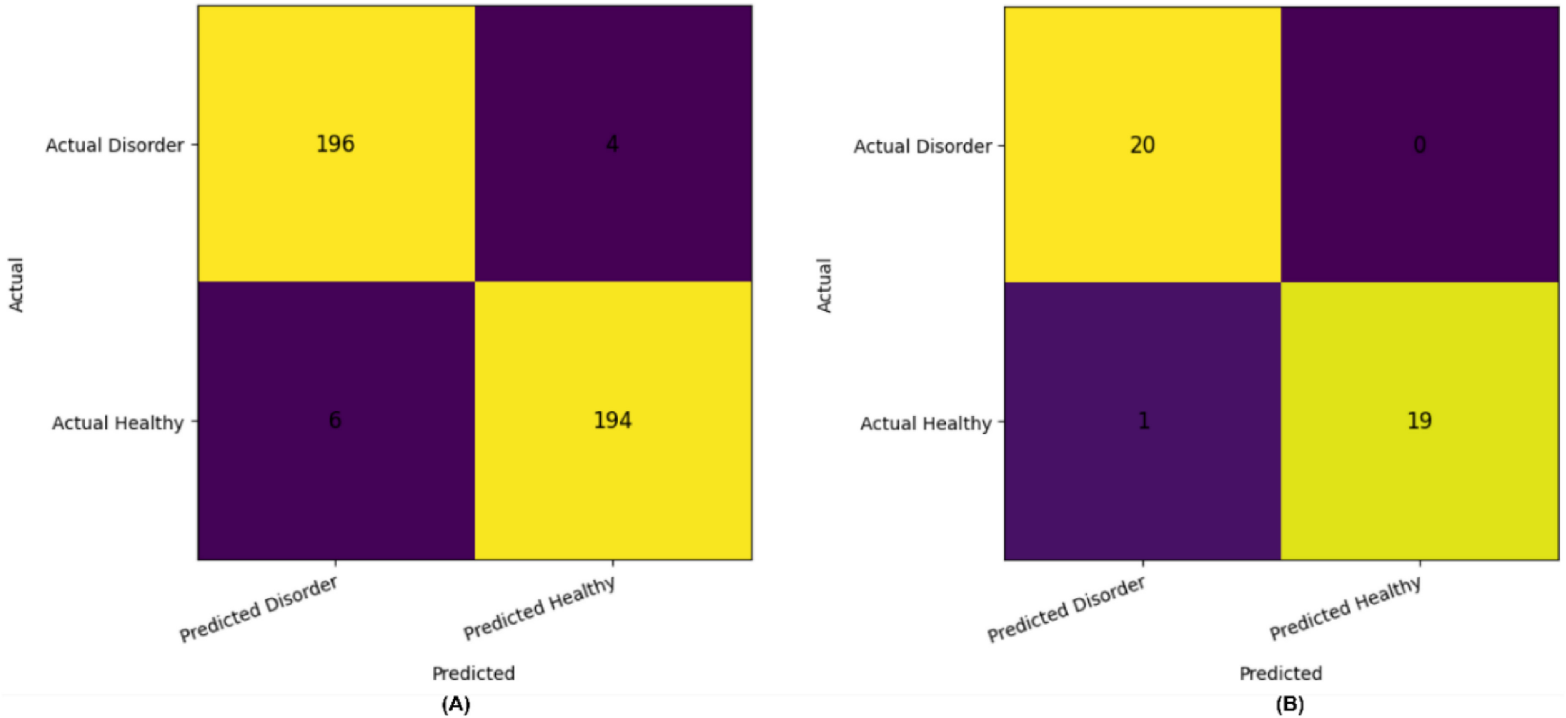
Confusion matrices of the proposed model on **(A)** SVD- test set **(B)** PD dataset.

Figure 5 represents confusion matrices for the SVD test set and the PD dataset. The confusion matrices show the classification performance of the proposed model during both internal and external validation. For instance, the confusion matrix for the SVD test set (Figure 5A) shows that the model correctly classifies 196 disorders and 194 healthy samples with no significant errors. This implies that the proposed model achieves balanced accuracy across both categories. On the other hand, in the PD dataset (Figure 5B), the model can identify all the 20 disorder cases and 19 out of the 20 healthy samples. Furthermore, the model’s stability across different datasets indicates its robustness to subject-independent data.

The computational efficiency of the proposed model compared to several benchmark CNN- and transformer-based approaches is presented in Table 7, in terms of parameters, floating-point operations, and inference time. The proposed approach balances computational complexity with 5.2 million parameters and 3.8 GFLOPs, achieving high efficiency and better performance than most other approaches considered in this study. Although MobileNetV3 shows lower computational costs, this approach has lower representation power, resulting in lower classification performance. On the other hand, the transformer-based ViT and Performer approaches have significantly higher computational costs, shown through greater FLOPs and inference time. EfficientNet-B7 also shows high computational complexity, despite the lack of improved performance. The proposed approach achieves computational efficiency through light-weight convolutions and optimized attention mechanisms, resulting in fast inference (18.5 ms) and stable computation.

**TABLE 7:** Findings of uncertainty analysis with number of parameters and FLOPs.

Classifier	Parameters (million)	FLOPs (GFLOPs)	Inference time (ms)
Proposed model	5.2	3.8	18.5
MobileNet V3	4.6	3.2	19.2
RegNetX	6.8	4.9	22.8
EfficientNet B7	9.5	7.2	35.4
Linformer	7.4	5.6	26.7
ViT	10.8	8.1	38.2
Performer	8.2	6.3	29.5

**FIGURE 6:**
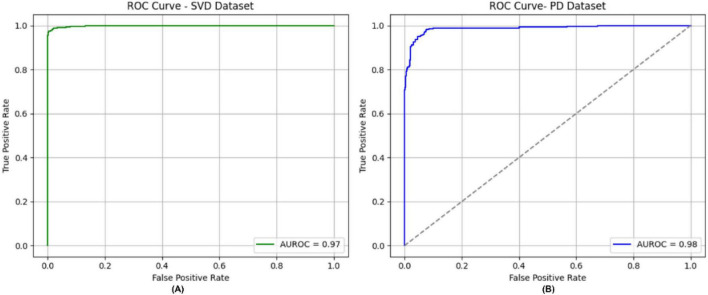
Findings of AUROC analysis. **(A)** SVD-test set. **(B)** PD dataset.

Figure 6 illustrates the remarkable performance of the SD detection model. The model achieves high sensitivity and specificity on SVD and PD datasets, maintaining robust classification capabilities across diverse clinical speech datasets. The high area under the receiver operating characteristic (ROC) curve (AUROC) underscores the diagnostic reliability of the proposed model across distinct pathological speech conditions. The integration of CNN-based local feature extraction with transformer-based global context modeling enables the model to differentiate the SD and healthy speech samples with optimal accuracy. Additionally, using the CaiT-based feature refinement leads to precise and interpretable predictions.

**FIGURE 7:**
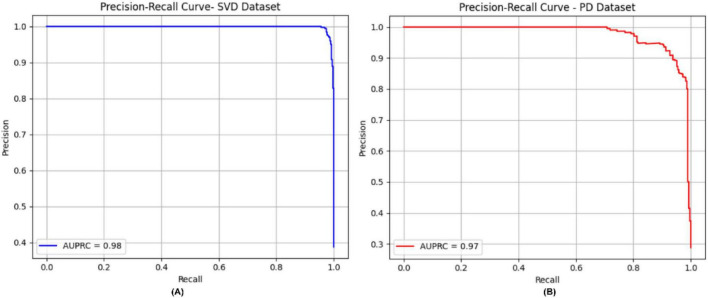
Findings of AUPRC analysis. **(A)** SVD-test set. **(B)** PD dataset.

Figure 7 illustrates the area under the precision-recall curve (AUPRC) for the SVD and PD datasets, highlighting the model’s discriminative capability. The findings underscore the model’s potential to deliver reliable predictions across different SD and recording conditions, confirming its suitability for real-world deployment. The high AUPRC value can be attributed to efficient hyperparameter tuning via BOHB. In addition, the proposed data pre-processing and augmentation technique enable the proposed model to focus on critical SD and healthy speech patterns.

Table 8 presents the additional external validation results on the VOICED dataset. Compared with the SVD and PD datasets, performance on VOICED is slightly lower due to heterogeneous pathological voice conditions and cross-dataset recording variability. Nevertheless, the proposed model achieves the highest accuracy of 95.80%, with balanced recall, precision, F1-score, and specificity. The lower RMSE value and statistically significant McNemar’s test results against baseline models confirm that the proposed channel-wise adaptive fusion and CaiT-based refinement improve generalization under heterogeneous pathological speech conditions.

**TABLE 8:** Comparison of classification performance: proposed model vs. pre-trained CNN and ViT models (VOICED dataset).

Classifier	Accuracy (%)	Precision (%)	Recall (%)	F1-score (%)	Specificity (%)	RMSE	Mean ± Std (%)	95% CI (Accuracy)	McNemar’s *p*-value
Proposed model	95.80	95.10	96.40	95.74	95.20	0.205	95.80 ± 2.10	93.85–97.75	–
MobileNet V3	91.85	90.40	89.75	90.07	90.95	0.286	91.85 ± 2.80	89.20–94.50	0.012
RegNetX	92.60	91.25	90.80	91.02	91.40	0.272	92.60 ± 2.65	90.05–95.15	0.018
EfficientNet B7	93.20	92.10	91.65	91.87	92.00	0.261	93.20 ± 2.45	90.85–95.55	0.026
Linformer	92.95	91.80	91.20	91.50	91.75	0.266	92.95 ± 2.50	90.55–95.35	0.031
ViT	92.40	91.10	90.55	90.82	91.20	0.276	92.40 ± 2.70	89.80–95.00	0.021

**FIGURE 8:**
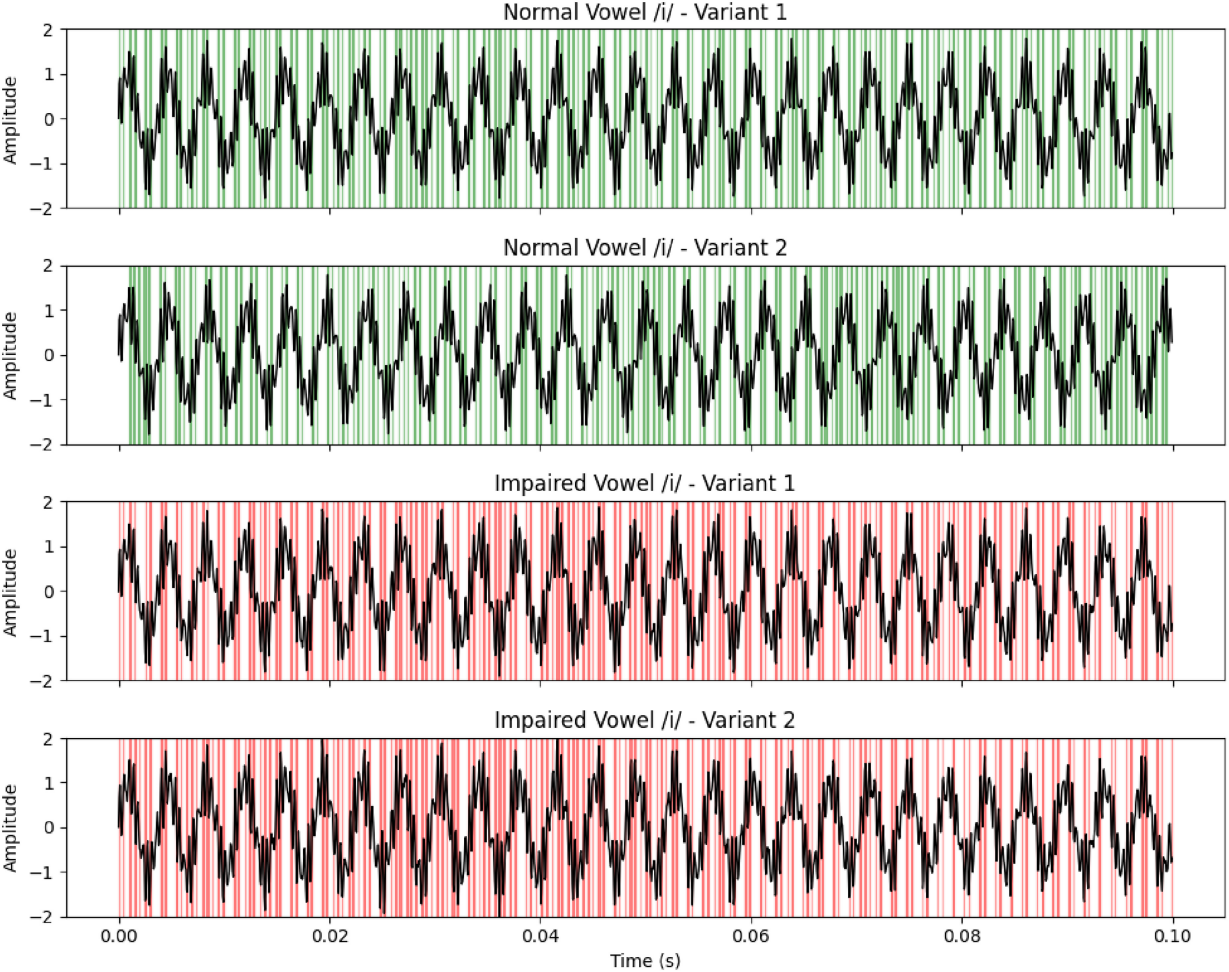
Sample outcomes of PD patient and healthy individuals speech analysis for vowel /i/ (PD dataset).

Through the use of waveform visualizations (Figure 8), an effective demonstration of the interpretability and diagnostic value of the proposed hybrid one-dimensional CNN–ViT model in the context of speech disorder identification is provided. The model’s attention and Grad-CAM algorithms highlighted temporal regions—green for healthy individuals and red for PD patients—as significant to its classification decisions, linking the model’s internal feature representations to interpretable acoustic events. For healthy individuals, the model emphasizes periodic, harmonically rich oscillations with stable amplitude. In the presence of intact neurological phonation, these patterns are acoustically associated with the controlled vocal fold vibrations. In contrast, for PD patients, the model focuses on regions with irregular amplitude modulations, pitch fluctuations, and distorted temporal structure, which are strongly related to phonatory instability, vocal tremor, and reduced neuromuscular control. Integrating local and global feature learning renders the model sensitive to fine-grained perturbations (jitter, shimmer) and long-range prosodic irregularities (rhythm instability, breath-voice timing). The model’s performance is greatly enhanced by its ability to detect and highlight acoustic signals associated with disorders.

**FIGURE 9:**
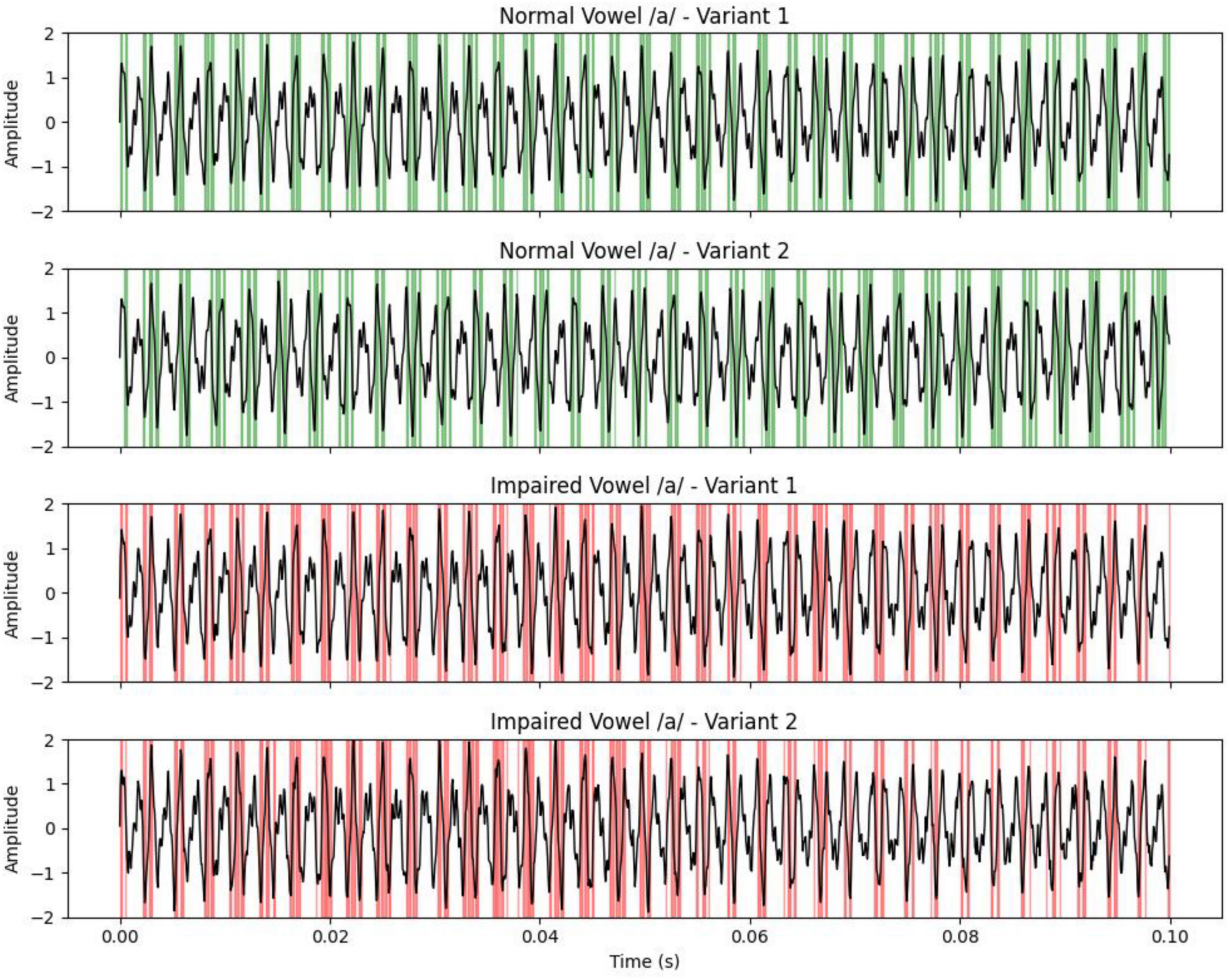
Sample outcomes of PD patient and healthy individuals speech analysis for vowel /a/ (PD dataset).

As a typical diagnostic indicator in voice evaluations, the waveform analysis in Figure 9 demonstrates the interpretability of the proposed model for the vowel /a/ phonation. The green-highlighted regions show regulated and sustained phonation with rhythmically consistent, symmetric oscillations and stable amplitude patterns in healthy individuals. The model’s focus on these areas indicates its recognition of voice regularity and harmonic structure as typical characteristics for differentiating healthy and SD audio waveforms. Overall, the interpretability increases transparency, improves clinician-model cooperation, and strengthens the model’s neurological voice disorder diagnostics decision-support functionality.

From the comparative analysis (Table 9), the proposed model proves to be highly accurate and consistent during internal and external evaluations, with an accuracy score of 97.50%. Although some methods, such as those using the XGBoost model, attain slightly higher accuracy, their efficiency is hindered by significantly larger parameter counts (12.3 million). Besides, these methods do not offer end-to-end feature learning and interpretation capabilities. Conversely, the proposed model maintains high performance efficiency despite having a smaller number of parameters (5.2 million). In contrast, traditional machine learning models such as SVMs and RFs demonstrate poor performance and limited ability to learn complex temporal-spectral features. Similarly, CNNs and ViTs either conduct static feature extraction or fail to adaptively integrate feature information. The proposed model addresses these challenges by integrating adaptive features and refining channels using CaiT modules.

**TABLE 9:** Comparison of classification performance: proposed model vs. existing state-of-the-art approaches.

Approaches	Classification approach	Performance
Proposed model	Fully connected layer with grad-CAM and attention visualization	Accuracy:97.50% Precision:97.03% Recall: 98.00% F1-score:97.51% Specificity:97.00% Parameters: 5.2 Millions
Proposed model	Fully connected layer with grad-CAM and attention visualization	Accuracy:97.50% Precision:95.24% Recall:100.00% F1-score: 97.56% Specificity:95.00% Parameters:5.2 Millions
Sait et al. (41)	XGBoost with SHAP values	Accuracy:98.90% Precision:98.00% Recall:98.10% F1-score:98.10% Specificity:97.80% Parameters:12.3 Millions
Abdullah et al. (42)	Fully connected layer with sigmoid function	Accuracy: 95.00% Precision: 98.00%
Ribas et al. (43)	Random forest	Accuracy: 93.90%
Hegde et al. (44)	Fully connected layer with sigmoid function	Accuracy: 90.00% Precision: 90.30% F1-score: 83.7%
Karan et al. (45)	SVM	Accuracy: 90.00%
Rana et al. (46)	SVM and ANN	SVM-Accuracy: 87.00% Artificial Neural Network- Accuracy: 96.7%
Hoq et al. (47)	SVM	Accuracy: 93.5% F1-score: 95.1%
Ali et al. (48)	SVM	Accuracy: 95.00% Sensitivity: 100.00% Specificity: 90.00%
Faragó et al. (49)	Fully connected layer with sigmoid function	Accuracy: 96.00%
Rahmatallah et al. (50)	Fully connected layer with sigmoid function	AUROC: 0.95 – 0.97
Jeong et al. (51)	Fully connected layer with sigmoid function	Accuracy: 92.15% Sensitivity: 91.53% Specificity: 92.79% AUROC: 0.97
Chen et al. (52)	Fully connected layer with sigmoid function	Accuracy: 95.56% F1-score: 95.47% AUROC: 0.99
Quamar et al. (53)	BiLSTM with sigmoid function	Accuracy: 97.00% F1-score: 97.00% AUROC: 0.95
Jayan et al. (63)	ViT backbone	Accuracy: 97.48% Recall: 97.47% F1-score: 97.47%
Poor et al. (64)	SWIN transformer backbone	AUROC: 85.3%

## 5 Discussion

The study presents novel contributions to feature extraction, feature refinement, and model interpretability that overcome critical limitations of existing SD detection frameworks. Using an end-to-end pipeline, the model identifies low- and high-level features relevant to disorder characterization without requiring manual intervention. By effectively leveraging one-dimensional CNNs and ViT’s self-attention mechanisms, the model enables a deeper understanding of SD context, which can help clinicians and speech-language pathologists detect it at early stages. The introduction of adaptive fusion and CaiT-based feature refinement increases the model’s robustness across diverse speech patterns, thereby improving its generalization to unseen data. The experimental analysis emphasizes the importance of the proposed SD detection method, which achieves 97.50 accuracy on the SVD and PD datasets, respectively. Visualizing critical and healthy SD patterns helps clinicians better understand the temporal underpinnings of the predictions, thereby improving model transparency and trust.

The quantitative and expert-based interpretability assessment showed that the proposed model consistently focused on clinically meaningful temporal regions of the speech waveform. Grad-CAM achieved a high localization agreement with disorder-relevant waveform segments, with an average overlap score of 0.82, while attention maps demonstrated stable focus consistency across pathological samples. Clinical expert validation further confirmed the relevance of the highlighted regions, with 87% agreement between expert-identified abnormal segments and model-highlighted regions. In addition, the highlighted areas showed strong correlation with known pathological acoustic biomarkers, including irregular amplitude modulation, unstable phonation, abnormal pauses, reduced phonatory regularity, and articulation-related disruptions. The correlation between model-highlighted regions and clinically recognized acoustic abnormalities reached *r* = 0.74, indicating meaningful alignment between model explanations and pathological speech characteristics. These findings demonstrate that the model does not rely solely on hidden statistical patterns but attends to clinically interpretable speech regions.

Compared with conventional spectrogram-based approaches, the proposed method operates on raw voice samples, thereby reducing the complexity of data preprocessing pipelines and initiating new avenues for flexible augmentation strategies. Micro-temporal anomalies such as tremor bursts, jitter irregularities, and shimmer variations, detected using one-dimensional convolutional filters, are identified, assisting clinicians in recognizing SD at early stages. The lightweight ViT module uses a self-attention mechanism that complements the CNN’s feature-extraction approach, enabling detection of broader prosodic shifts, breadth-phenome coupling disruptions, and rhythm disturbances associated with neuro-motor conditions. The proposed adaptive feature-fusion mechanism promotes reweighting of features from CNNs and ViTs, supporting accurate classification of the speech signal and outperforming traditional machine learning methods and modern deep neural networks based on the spectrogram. Experimental results show that this adaptive fusion can improve classification performance, thereby addressing overfitting in moderate-sized datasets.

During model implementation, several limitations and challenges were identified. Using raw voice samples simplifies the data preprocessing pipeline; however, this approach introduces new issues with model convergence and stability. Training based on raw audio waveforms makes the model sensitive to initial hyperparameter settings, such as the learning rate and filter initialization, thus requiring longer tuning cycles. The lack of frequency decomposition decreases the ability of the model to identify phonetic overlapping events. While the adaptive fusion gate enhances the generalization, it adds a layer of complexity to the training dynamics. By dynamically balancing two streams of information per sample, the probability of optimization instability is increased, especially during the early stages of training. Without properly scheduled training, the model can be overly dependent on either the CNN or Transformer stream. The development of strong techniques or gate regularizers is therefore critical in order to perform better generalization.

The model performed well on the multi-disease dataset. However, the voice samples were recorded in a medium-controlled environment with smartphone-grade microphones. Improving the model’s capacity to handle a wider range of real-time environments is a challenging task. By leveraging substantial data augmentation techniques, the proposed model’s generalization performance can be improved. Manifestations of speech impairments often vary across languages and cultural contexts; hence, additional validation studies across a wide range of linguistic communities are imperative. The future research should involve cross-language transfer learning as well as dialectal diversity. Though the explainability approach produced meaningful visuals, saliency maps cannot be guaranteed to establish causality without the knowledge of the subject studied, and some attributions may be misinterpreted. Thus, a compelling model-clinical credibility correlation may be built through inherently interpretable model architectures. The use of multimodal inputs such as breathing patterns, facial movements, or accelerometry data has the potential to greatly improve diagnostic accuracy in the early stages. Employing self-supervised pre-training on large amounts of raw audio corpora can improve representation learning, thereby reducing reliance on labeled clinical datasets. To overcome privacy and scalability issues, federated learning is required to train models securely and in a decentralized manner across clinics. Finally, longitudinal studies may enable prediction of disease progression and static diagnoses, ultimately fostering proactive treatment strategies.

## 6 Conclusion

This study presents a unique SD detection framework that demonstrates superior performance, interpretability, and computational efficiency. By leveraging raw audio waveforms, the proposed model mitigates the need for multi-stage audio processing pipeline and handcrafted acoustic features. Using an adaptive fusion and CaiT feature refinement, the model successfully integrate CNN and ViT features, enabling precise detection of relevant temporal and contextual patterns associated with speech impairments. The novel contribution of this study is the development of a lightweight architecture for detecting SD in the resource-constrained environment. The model achieves significant performance on standard classification metrics and generalizes well across two benchmark datasets, with limited number of parameters and FLOPs. In addition, integrating Grad-CAM and attention-based visualization enhances the model’s interpretability by pinpointing the crucial temporal segments responsible for the prediction, thereby rendering meaningful outcomes. Although the model demonstrates robust performance across datasets, it has several limitations. Reliance on raw audio waveforms may pose challenges in settings with low-fidelity recordings. The use of CNNs and ViTs for feature extraction requires substantial training time and additional hyperparameter tuning for complex datasets. Future studies should focus on model improvement in terms of multi-lingual processing and dialectal diversity. Using self-supervised pre-training algorithms and federated learning can improve model generalization and address challenges related to privacy and scalability.

## Data Availability

The original contributions presented in this study are included in the article/Supplementary material, further inquiries can be directed to the corresponding author.
