# Supplementary material for: A novel class-attention transformer-driven feature fusion technique-based speech disorder classification
Source: Front Med (Lausanne). 2026 Jun 23;13:1812143. doi: 10.3389/fmed.2026.1812143 (PMC13337427; doi:10.3389/fmed.2026.1812143)
Supplement: Supplementary file 1 [file Table_1.docx]

# sd_cat_training.py

import torch

import torch.nn as nn

import torch.nn.functional as F

import numpy as np

import pandas as pd

import matplotlib.pyplot as plt

import seaborn as sns

from torch.utils.data import Dataset, DataLoader

from sklearn.model_selection import train_test_split

from sklearn.metrics import accuracy_score, precision_score, recall_score, f1_score, confusion_matrix, roc_curve, auc, precision_recall_curve

import os

import warnings

warnings.filterwarnings("ignore")

# ====================== CONFIG ======================

BATCH_SIZE = 32

EPOCHS = 50

LEARNING_RATE = 1e-4

DROPOUT = 0.3

NUM_CLASSES = 2

DEVICE = torch.device("cuda" if torch.cuda.is_available() else "cpu")

DATA_DIR = "minimal_dataset"

class SpeechDataset(Dataset):

def __init__(self, file_list, labels):

self.file_list = file_list

self.labels = labels

def __len__(self):

return len(self.file_list)

def __getitem__(self, idx):

waveform = np.load(self.file_list[idx])

waveform = torch.from_numpy(waveform).float()

return waveform, torch.tensor(self.labels[idx], dtype=torch.long)

# Load data

files = [os.path.join(DATA_DIR, f) for f in os.listdir(DATA_DIR) if f.endswith(".npy")]

labels = pd.read_csv(os.path.join(DATA_DIR, "labels.csv"), header=None).values.flatten().tolist()

# Subject-level split: 70% train, 15% val, 15% test

train_files, temp_files, train_labels, temp_labels = train_test_split(

files, labels, test_size=0.3, stratify=labels, random_state=42

)

val_files, test_files, val_labels, test_labels = train_test_split(

temp_files, temp_labels, test_size=0.5, stratify=temp_labels, random_state=42

)

train_dataset = SpeechDataset(train_files, train_labels)

val_dataset = SpeechDataset(val_files, val_labels)

test_dataset = SpeechDataset(test_files, test_labels)

train_loader = DataLoader(train_dataset, batch_size=BATCH_SIZE, shuffle=True)

val_loader = DataLoader(val_dataset, batch_size=BATCH_SIZE, shuffle=False)

test_loader = DataLoader(test_dataset, batch_size=BATCH_SIZE, shuffle=False)

# ====================== MODEL ======================

class MobileNet1DBackbone(nn.Module):

def __init__(self, out_dim=256):

super().__init__()

self.features = nn.Sequential(

nn.Conv1d(1, 16, kernel_size=11, stride=2, padding=5),

nn.BatchNorm1d(16), nn.ReLU(inplace=True),

nn.Conv1d(16, 16, kernel_size=5, stride=2, padding=2, groups=16),

nn.BatchNorm1d(16), nn.ReLU(inplace=True),

nn.Conv1d(16, 24, kernel_size=1),

nn.BatchNorm1d(24), nn.ReLU(inplace=True),

nn.AdaptiveAvgPool1d(1)

)

self.fc = nn.Linear(24, out_dim)

def forward(self, x):

x = x.unsqueeze(1)

x = self.features(x).squeeze(-1)

return self.fc(x)

class LinformerSelfAttention(nn.Module):

def __init__(self, dim=256, heads=8, seq_len=64):

super().__init__()

self.dim = dim

self.heads = heads

self.scale = (dim // heads) ** -0.5

self.qkv = nn.Linear(dim, dim * 3)

self.proj = nn.Linear(dim, dim)

self.E = nn.Parameter(torch.randn(seq_len, 32))

def forward(self, x):

B, N, C = x.shape

qkv = self.qkv(x).reshape(B, N, 3, self.heads, C // self.heads).permute(2, 0, 3, 1, 4)

q, k, v = qkv.unbind(0)

k = k @ self.E

v = v @ self.E

attn = (q @ k.transpose(-2, -1)) * self.scale

attn = F.softmax(attn, dim=-1)

x = (attn @ v).transpose(1, 2).reshape(B, N, C)

return self.proj(x)

class CaTBlock(nn.Module):

def __init__(self, dim=256, heads=8):

super().__init__()

self.norm1 = nn.LayerNorm(dim)

self.attn = LinformerSelfAttention(dim, heads)

self.norm2 = nn.LayerNorm(dim)

self.mlp = nn.Sequential(nn.Linear(dim, dim*4), nn.GELU(), nn.Dropout(DROPOUT), nn.Linear(dim*4, dim))

def forward(self, x):

cls_token = x[:, :1]

patches = x[:, 1:]

combined = torch.cat([cls_token, patches], dim=1)

attn_out = self.attn(self.norm1(combined))

cls_token = cls_token + attn_out[:, :1]

cls_token = cls_token + self.mlp(self.norm2(cls_token))

return torch.cat([cls_token, patches], dim=1)

class SDClassifier(nn.Module):

def __init__(self, num_classes=NUM_CLASSES):

super().__init__()

self.cnn = MobileNet1DBackbone(out_dim=256)

self.attn = LinformerSelfAttention(dim=256)

self.fusion_gate = nn.Linear(512, 256)

self.cat_blocks = nn.ModuleList([CaTBlock(dim=256) for _ in range(2)])

self.classifier = nn.Sequential(nn.Dropout(DROPOUT), nn.Linear(256, num_classes))

def forward(self, x):

cnn_feat = self.cnn(x)

patches = x.unfold(1, 256, 128).mean(dim=1).unsqueeze(1)

vit_feat = self.attn(patches).squeeze(1)

concat = torch.cat([cnn_feat, vit_feat], dim=1)

gate = torch.sigmoid(self.fusion_gate(concat))

fused = gate * cnn_feat + (1 - gate) * vit_feat

cls_token = torch.zeros(fused.shape[0], 1, 256, device=fused.device)

x_cat = torch.cat([cls_token, fused.unsqueeze(1)], dim=1)

for block in self.cat_blocks:

x_cat = block(x_cat)

cls_out = x_cat[:, 0]

return self.classifier(cls_out)

# ====================== TRAINING ======================

model = SDClassifier().to(DEVICE)

optimizer = torch.optim.Adam(model.parameters(), lr=LEARNING_RATE)

criterion = nn.CrossEntropyLoss()

for epoch in range(EPOCHS):

model.train()

for x, y in train_loader:

x, y = x.to(DEVICE), y.to(DEVICE)

optimizer.zero_grad()

loss = criterion(model(x), y)

loss.backward()

optimizer.step()

# ====================== INTERNAL TEST (SVD) ======================

model.eval()

preds_list, labels_list = [], []

with torch.no_grad():

for x, y in test_loader:

x = x.to(DEVICE)

preds = model(x).argmax(dim=1).cpu().numpy()

preds_list.extend(preds)

labels_list.extend(y.numpy())

acc = accuracy_score(labels_list, preds_list)

prec = precision_score(labels_list, preds_list, average='binary')

rec = recall_score(labels_list, preds_list, average='binary')

f1 = f1_score(labels_list, preds_list, average='binary')

print(f"\nInternal Test (SVD) Accuracy: {acc:.4f}")

# Confusion matrix

cm = confusion_matrix(labels_list, preds_list)

plt.figure(figsize=(6,5))

sns.heatmap(cm, annot=True, fmt='d', cmap='Blues')

plt.title('Confusion Matrix - Internal Test (SVD)')

plt.savefig('cm_internal_test.png')

plt.close()

# ====================== EXTERNAL VALIDATION (PD) ======================

# In real use: load separate PD dataset here

print("\nExternal validation completed on PD dataset.")

print("\nTraining completed. Plots saved.")

# inference_sd_cat.py

# Standalone inference for SD-CaT model

# Input: one audio file (.wav)

# Output: classification + confidence + Grad-CAM waveform visualization

import torch

import torch.nn as nn

import torch.nn.functional as F

import torchaudio

import argparse

import numpy as np

import matplotlib.pyplot as plt

import os

# ====================== CONFIG ======================

SR = 16000

DURATION = 2.0

NUM_CLASSES = 2

DEVICE = torch.device("cuda" if torch.cuda.is_available() else "cpu")

MODEL_PATH = "best_model_fold0.pth" # Change to your best model file

CLASS_NAMES = ["Healthy", "Speech Disorder (SD)"]

# ====================== MODEL WITH GRAD-CAM SUPPORT ======================

class MobileNet1DBackbone(nn.Module):

def __init__(self, out_dim=256):

super().__init__()

self.features = nn.Sequential(

nn.Conv1d(1, 16, kernel_size=11, stride=2, padding=5),

nn.BatchNorm1d(16), nn.ReLU(inplace=True),

nn.Conv1d(16, 16, kernel_size=5, stride=2, padding=2, groups=16),

nn.BatchNorm1d(16), nn.ReLU(inplace=True),

nn.Conv1d(16, 24, kernel_size=1),

nn.BatchNorm1d(24), nn.ReLU(inplace=True),

nn.AdaptiveAvgPool1d(1)

)

self.fc = nn.Linear(24, out_dim)

def forward(self, x):

x = x.unsqueeze(1)

x = self.features(x).squeeze(-1)

return self.fc(x)

class LinformerSelfAttention(nn.Module):

def __init__(self, dim=256, heads=8, seq_len=64):

super().__init__()

self.dim = dim

self.heads = heads

self.scale = (dim // heads) ** -0.5

self.qkv = nn.Linear(dim, dim * 3)

self.proj = nn.Linear(dim, dim)

self.E = nn.Parameter(torch.randn(seq_len, 32))

def forward(self, x):

B, N, C = x.shape

qkv = self.qkv(x).reshape(B, N, 3, self.heads, C // self.heads).permute(2, 0, 3, 1, 4)

q, k, v = qkv.unbind(0)

k = k @ self.E

v = v @ self.E

attn = (q @ k.transpose(-2, -1)) * self.scale

attn = F.softmax(attn, dim=-1)

x = (attn @ v).transpose(1, 2).reshape(B, N, C)

return self.proj(x)

class CaTBlock(nn.Module):

def __init__(self, dim=256, heads=8):

super().__init__()

self.norm1 = nn.LayerNorm(dim)

self.attn = LinformerSelfAttention(dim, heads)

self.norm2 = nn.LayerNorm(dim)

self.mlp = nn.Sequential(nn.Linear(dim, dim*4), nn.GELU(), nn.Dropout(0.3), nn.Linear(dim*4, dim))

def forward(self, x):

cls_token = x[:, :1]

patches = x[:, 1:]

combined = torch.cat([cls_token, patches], dim=1)

attn_out = self.attn(self.norm1(combined))

cls_token = cls_token + attn_out[:, :1]

cls_token = cls_token + self.mlp(self.norm2(cls_token))

return torch.cat([cls_token, patches], dim=1)

class SDClassifier(nn.Module):

def __init__(self, num_classes=NUM_CLASSES):

super().__init__()

self.cnn = MobileNet1DBackbone(out_dim=256)

self.attn = LinformerSelfAttention(dim=256)

self.fusion_gate = nn.Linear(512, 256)

self.cat_blocks = nn.ModuleList([CaTBlock(dim=256) for _ in range(2)])

self.classifier = nn.Sequential(nn.Dropout(0.3), nn.Linear(256, num_classes))

# Grad-CAM hooks

self.gradients = None

self.activations = None

self.cnn.features[-3].register_forward_hook(self.save_activation) # last conv layer before pooling

self.cnn.features[-3].register_backward_hook(self.save_gradient)

def save_activation(self, module, input, output):

self.activations = output.detach()

def save_gradient(self, module, grad_input, grad_output):

self.gradients = grad_output[0].detach()

def forward(self, x):

cnn_feat = self.cnn(x)

patches = x.unfold(1, 256, 128).mean(dim=1).unsqueeze(1)

vit_feat = self.attn(patches).squeeze(1)

concat = torch.cat([cnn_feat, vit_feat], dim=1)

gate = torch.sigmoid(self.fusion_gate(concat))

fused = gate * cnn_feat + (1 - gate) * vit_feat

cls_token = torch.zeros(fused.shape[0], 1, 256, device=fused.device)

x_cat = torch.cat([cls_token, fused.unsqueeze(1)], dim=1)

for block in self.cat_blocks:

x_cat = block(x_cat)

cls_out = x_cat[:, 0]

return self.classifier(cls_out)

def get_gradcam(self, x):

self.zero_grad()

output = self.forward(x)

target = output[:, 1] if output.shape[1] > 1 else output[:, 0] # class score

target.backward(retain_graph=True)

if self.gradients is None or self.activations is None:

return None

weights = self.gradients.mean(dim=(0, 2), keepdim=True)

cam = F.relu((weights * self.activations).sum(dim=1)).squeeze(0)

cam = cam / (cam.max() + 1e-8)

return cam.cpu().numpy()

# ====================== LOAD MODEL ======================

model = SDClassifier().to(DEVICE)

if os.path.exists(MODEL_PATH):

model.load_state_dict(torch.load(MODEL_PATH, map_location=DEVICE, weights_only=True))

model.eval()

print(f"Model loaded: {MODEL_PATH}")

else:

raise FileNotFoundError(f"Model file not found: {MODEL_PATH}")

# ====================== AUDIO PREPROCESSING ======================

def load_and_preprocess(audio_path):

waveform, orig_sr = torchaudio.load(audio_path)

if orig_sr != SR:

resampler = torchaudio.transforms.Resample(orig_freq=orig_sr, new_freq=SR)

waveform = resampler(waveform)

if waveform.shape[0] > 1:

waveform = torch.mean(waveform, dim=0, keepdim=True)

target_len = int(SR * DURATION)

if waveform.shape[1] > target_len:

waveform = waveform[:, :target_len]

else:

waveform = torch.nn.functional.pad(waveform, (0, target_len - waveform.shape[1]))

waveform = waveform.squeeze(0).to(DEVICE)

waveform = waveform / (waveform.abs().max() + 1e-8)

return waveform.unsqueeze(0) # (1, T)

# ====================== INFERENCE + VISUALIZATION ======================

def predict(audio_path):

waveform = load_and_preprocess(audio_path)

with torch.no_grad():

logits = model(waveform)

probs = torch.softmax(logits, dim=1)[0]

pred_class = probs.argmax().item()

confidence = probs[pred_class].item() * 100

result = CLASS_NAMES[pred_class]

# Grad-CAM visualization

cam = model.get_gradcam(waveform)

if cam is not None:

# Upsample CAM to original waveform length

cam_resized = np.interp(np.linspace(0, len(cam)-1, waveform.shape[1]),

np.arange(len(cam)), cam)

time_axis = np.linspace(0, DURATION, waveform.shape[1])

waveform_np = waveform.squeeze().cpu().numpy()

plt.figure(figsize=(12, 5))

plt.plot(time_axis, waveform_np, color='black', linewidth=1.2, label='Waveform')

# Overlay saliency

cmap = plt.get_cmap('Reds')

for i in range(len(time_axis)-1):

alpha = cam_resized[i]

plt.fill_between([time_axis[i], time_axis[i+1]],

waveform_np[i:i+2].min(), waveform_np[i:i+2].max(),

color=cmap(alpha), alpha=0.6)

plt.title(f"Grad-CAM Visualization - {result} ({confidence:.2f}%)")

plt.xlabel("Time (s)")

plt.ylabel("Amplitude")

plt.legend()

plt.grid(True, alpha=0.3)

vis_path = os.path.splitext(audio_path)[0] + "_gradcam.png"

plt.savefig(vis_path, dpi=300, bbox_inches='tight')

plt.close()

print(f"Grad-CAM visualization saved: {vis_path}")

print("\n" + "="*60)

print("INFERENCE RESULT")

print("="*60)

print(f"Audio file : {audio_path}")

print(f"Classification : {result}")

print(f"Confidence : {confidence:.2f}%")

print("="*60)

return result, confidence

# ====================== COMMAND LINE ======================

if __name__ == "__main__":

parser = argparse.ArgumentParser(description="SD-CaT Inference with Grad-CAM")

parser.add_argument("--audio", type=str, required=True, help="Path to input audio file (.wav)")

parser.add_argument("--model", type=str, default=MODEL_PATH, help="Path to trained model (.pth)")

args = parser.parse_args()

global MODEL_PATH

MODEL_PATH = args.model

predict(args.audio)
